# A green approach to improve antibacterial properties of PVC/PVDF film doped by silver nanoparticles via nanosecond laser ablation for wound healing application

**DOI:** 10.1038/s41598-024-78841-1

**Published:** 2024-11-13

**Authors:** S. S. El-Bahnasy, Mohamed Khalaf, D. M. Ayad, A. A. Menazea

**Affiliations:** 1 Chemistry Department, Faculty of Science, Mansoura University, Mansoura, 35516 Egypt; 2 Department of Physics, Faculty of Science, Suez University, Suez, 43518 Egypt; 3 Spectroscopy Department, Physics Research Institute, National Research Centre, Dokki, 12622 Giza Egypt

**Keywords:** PVC/PVDF, Silver nanoparticles, Laser ablation, Antibacterial activity, Biophysics, Nanoscience and technology

## Abstract

Nanocomposite films of (30% PVC/70% PVDF) blend containing silver nanoparticles were synthesized via pulsed laser ablation route (PLA). Changes in physical characterization of PVC/PVDF blend before and after the incorporation of AgNPs have been studied. FTIR results confirms the interaction between AgNPs and PVC/PVDF. XRD results obtain the existence of peak at 38.42̊ in sample PVC/PVDF/Ag4 which confirm the embedded AgNPs in the high concentration. SEM photos confirm the distribution of silver nanoparticles on the surface of the sample in spherical shape which approved the dispersion of silver nanoparticles in PVC/PVDF blend. The inhibitory zone diameters observed for PVC/PVDF/Ag4 against *Escherichia coli*, *Klebsiella pneumonia*, *Staphylococcus aureus*, and *Bacillus cereus* were recorded as 11 ± 1, 10 ± 1, 15.7 ± 0.6, and 17.7 ± 0.6, respectively. PVC/PVDF/AgNPs nanocomposite film could be suggested for biomedical applications such as wound healing products.

## Introduction

A great deal of research has been done on nanoparticles due to their special physicochemical qualities, which include antibacterial, catalytic, optical, electrical, and magnetic capabilities^1^. Metal nanoparticles exhibit manifested size-related Physico-chemical properties different from their bulk counterpart^2^. MNPs are used in cosmetics, inks, catalysis, medicine, medical imaging, optics, environmental remediation, and renewable energies^3,4^. Noble metal NPs have impressive functional properties such as extensive coloration, antimicrobial properties, magnetic properties, and catalytic properties, thus their thin films are an intensive area of research^5,6^.

The laser is a system which, through an optical amplification mechanism, produces a beam of coherent light^7^. Laser ablation is an alternative physical process used for the preparation of nanoparticles under a wide extent of the condition^8^. The most effective physical method used for manufacturing the nanoparticles is PLAL^9,10^. PLAL is a simple and flexible physical process used for the production of nano-colloids compared to the other conventional method^11,12^. Through PLAL process, we can get non-toxic nanoparticles with desired shape and size compared to the other conventional route^13,14^.

AgNPs are consider one of most popular metal nanoparticles due to their biological, chemical, and physical properties^15,16^. AgNPs have a large interest in noble metal NPs study as they are used in pretending the relevance and diversity of the area of NPs in general. AgNPs have been a great useful resource due to their novel magnetic, chemical, physical, optical, and electrical properties^17^. AgNPs is extensively used in many medical and biological fields^18^. In order to act as antibacterial agents, silver and its ions interact with protein molecules either inside or outside of bacterial cell membranes and bind to the membrane itself. This limits the ability of DNA molecules to replicate, which negatively affects bacterial viability^19^. As a result, the role of commercially available silver-based treatments including topical ointments, bandages, and gels in enhancing public health care has grown. Silver-based compounds were found to have low washing resistance, making it challenging to maintain ideal release levels^20^.

Polymer attracts the curiosity of several scientists within the last decades. Polymers are flexible, recyclable, mouldable, and lightweight. In addition, doping polymeric matrices with nanoparticles may result in new applications^21–26^. Polyvinylidene fluoride (PVDF) was used due to its piezoelectric and pyroelectric characterization. Its highly non-reactive thermoplastic polymer with chemical formula [(C_2_H_2_F_2_)_n_]. β-phase responsible for its piezoelectric properties^21^. The higher rearrangement of molecular chain in β-phase of PVDF due to their net dipole moment. PVDF is used in numerous utilizations because of their high piezo-electricity, mechanical strength, resistance flexibility, and excellent dielectric characterization^27^. In addition, it has a good melting viscosity that makes it suitable for use in many purposes without the use of additional stabilizers or chemicals. Only polar organic materials can make it soluble^28^. Polyvinyl chloride (PVC) has an amorphous nature, with a chemical formula [(C2H3Cl)_n_], which has a remarkable properties and excellent processability with low cost. PVC has massive commercial and industrial application due to their characterization. Its compatibility and miscibility by other inorganic materials can be altered to produce flexible composites with varying mechanical and electrical characterization which could be employed in various utilization^29,30^. PVC is an excellent insulating material that is resistant to all inorganic compounds as well as rotting from chemicals, shock, and abrasion^31^. Both its mechanical and physical qualities are good. PVC can be used for a variety of things, including building materials, household items, and medical supplies^32^.

Herin, our paper is aims to develop a PVC/PVDF/AgNPs nanocomposite through a laser ablation route, incorporating varying concentrations of AgNPs. This study not only focuses on synthesizing the nanocomposite but also delves into investigating its physical characterization and antibacterial activity. By combining unique fabrication techniques with a detailed analysis of material characteristics and functional attributes, this research contributes to advancing the understanding and potential applications of nanocomposite materials in biomedical contexts, particularly in the realm of wound healing products. 

## Experimental work

### Materials

PVC with (Mw ≈ 500k Da) and PVDF with (Mw ≈ 543k Da) have been bought from Arcos. High pure (99.999%) silver plate has been purchased from Sigma Aldrich.

### Fabrication of PVC/PVDF blend solution

A pure blend of PVC/PVDF (70%/30%) without silver nanoparticles (AgNPs) was prepared. Tetrahydrofuran (THF) was used to dissolve the polymeric matrix with constant stirring for approximately 4 h until a viscous solution is formed.

### Fabrication of PVC/PVDF/AgNPs nanocomposite via PLAL route

AgNPs have been synthesized via laser ablation process. Nd: YAG nanosecond laser with 1064 nm wavelength has been utilized as a source of ablation technique. Silver plate has been put in the bottom of beaker filled by 20 ml of the previous prepared PVC/PVDF solution. The laser beam was incident vertically on the silver plate by the convex lens in order to obtain laser ablation on surface. Repeat these steps by changing the laser ablation time (5, 10, 20, and 30 min) in order to obtain different contents of AgNPs. The composition of five samples of PVC/PVDF/AgNPs nanocomposites with contents of AgNPs has been mentioned in Table 1. The final viscous solution was cast onto clean glass petri dishes and dried in an oven at 50° for 48 h.

**Table 1:** Sample composition PVC/PVDF/AgNPs nanocomposites with different ratios of silver nanoparticles.

Sample	Laser ablation time (min)	Content of AgNPs (g/L)
PVC/PVDF	−	−
PVC/PVDF/Ag1	5	0.049
PVC/PVDF/Ag2	10	0.087
PVC/PVDF/Ag3	20	0.112
PVC/PVDF/Ag4	30	0.143

### Characterization techniques

XRD was investigated by (Schimadzu 7000, Japan) occupied with Cu-Kα radiation (λ = 0.154060 nm) at 30 kV and 30 mA. FT-IR spectra of the fabricated films have been investigated within the wave number range 400–4000 cm^− 1^ using a spectrometer type (Jasco, 6100, Japan). UV–Visible spectroscopy analysis was performed by JASCO (V-570) double beam spectrophotometer in the wavelength region of 200–1000 nm. Morphological descriptions of the synthesized PVC/PVDF/AgNPs nanocomposites films were performed via FE-SEM type (Quanta FEG 250, USA).

### The antibacterial activity

The antibacterial activity of various compounds was assessed using the agar well diffusion method against a range of bacterial and fungal strains. Tested against *Escherichia coli*, *Klebsiella pneumonia*, *Staphylococcus aureus*, and *Bacillus Subtilis*. The compounds have been evaluated at a concentration of 15 mg/ml on nutrient agar or Sabouraud dextrose agar. Ampicillin, Gentamicin, and Nystatin served as standard drugs for bacteria and fungi. Sterile media was poured into Petri dishes, inoculated with microbial suspensions, and wells were filled with the test compounds. After incubation at 37 °C for 24 h, zones of inhibition were measured. Statistical analysis was conducted using ANOVA and Duncan multiple comparisons test in SPSS, with significance levels set at *p* < 0.05, *p* < 0.01, and *p* < 0.001 denoting different levels of statistical significance^33^. The analysis method used to assess the antibacterial performance of PVC/PVDF/Ag composites involved a One-Way Analysis of Variance (ANOVA). This statistical test was employed to determine if there were significant differences in the antimicrobial efficacy among the different material compositions (PVC/PVDF/Ag3, PVC/PVDF/Ag4) and standard antibiotic control. The ANOVA test evaluates the variance within each group and between groups to assess if the observed differences in their means are statistically significant. In this case, the antibacterial experiment was conducted three times for all the prepared samples, with each experiment resulting in slight variations around the mean. These replicated values provided enough data points to perform the ANOVA, which helps account for experimental variability and ensures the results are statistically robust. By analyzing the variance across the replicates, ANOVA enabled the identification of significant differences in antimicrobial activity between the Ag-modified materials and the control, confirming the enhanced efficacy of silver-incorporated composites.

### The cell viability test

To assess the survivability of cells seeded onto the membranes, the human osteoblast cell line HFB4 has been grown in Dulbecco’s modified Eagle’s medium (DMEM, Gibco) at 37 °C and 5% CO_2_. Cells inoculated at a density of 5 × 10^2^ cells/cm^2^ were cultivated on the fibers in 96-well plates. Following a three-day incubation at 37 °C, the medium was discarded, and MTT (3-(4,5-dimethylthiazol-2-yl)-2,5-diphenyltetrazolium bromide) was administered to each well, subsequently allowing for the assessment of cell viability using an optical analyzer.

## Results and discussion

### X-ray diffraction (XRD)

**Fig. 1:**
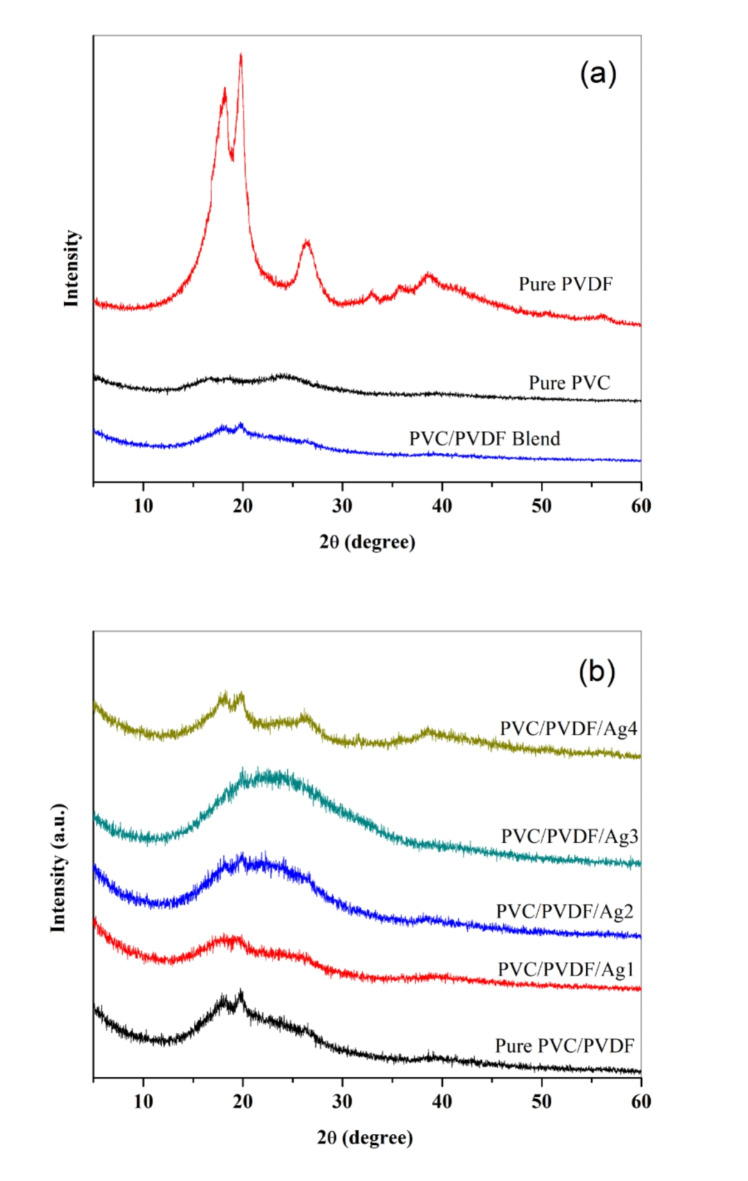
XRD patterns of (**a**) pure PVC, pure PVDF, and PVC/PVDF blend; (**b**) PVC/PVDF blend and PVC/PVDF with different ratios of silver nanoparticles.

XRD pattern showed the characterisation and amorphous nature for PVC while in PVDF showed semicrystalline nature. As shown in Fig. 1a, pure PVC the spectrum showed at two diffraction peak at 2θ = 16.84° and 24.51°^30,34^. The XRD pattern for pure PVDF correspond to the 2θ values at 18.27°, 19.52°, 26.47°, 32.89°, 35.74°, 38.58° and 56.23°. The crystalline structure of pure PVDF could be classified into α, β, γ phase based on the shape of crystallization^35^.

XRD of pure PVC/PVDF blend obtain the semi-crystalline nature as the main hallow peak centered at 2θ = 17.74° and 19.71°. The fundamental peaks of PVC have been completely disappeared in all the synthesized samples because of the complete blending of PVDF with PVC as shown in Fig. 1b. For PVC/PVDF/AgNPs samples, the peak observed at 2θ = 18.27° for pure PVDF disappear in the pure blend resulted from the addition of PVC which reduces the long-range order in PVDF. The peak observed at 2θ = 17.74° belonging to -phase crystal^36^ and β-phase obtained at peak of 2θ = 19.71^34^. After addition AgNPs to the blend, the marked peak at 38.42°^37^ implies the existence of the AgNPs within PVC/PVDF matrix. This result illustrates that AgNPs can cause structural change in PVC/PVDF matrix. By raising the contents of AgNPs in PVC/PVDF matrix, it causes change in the intensity and broadness of the unique peak. The intensity of this peak is decreasing with raising concentration of AgNPs, this decreasing approved a reduce in the degree of crystallinity and that proved the complete interaction between AgNPs and PVC/PVDF matrix. The absence of this peak in the other concentration may be due to the low doping of AgNPs in PVC/PVDF matrix. The arrangement of the polymeric chains is confirmed by the degree of the crystallinity. Adding silver nanoparticles to the blend lead to increase the amorphous nature of the blend that led to increase conductivity. By comparing the conductivity between polymer blend and polymer nanocomposite, the good conductivity obtained through polymer nanocomposite.

The crystalline size of AgNPs was calculated by Debye- Scherrer’s formula^26,30,38–41^:

1 $${\text{Size }} = {\text{ K}}\lambda /\beta \text{Cos} {\text{ }}\theta.$$

According the equation, the average size of doped AgNPs in PVC/PVDF at 2θ = 38.42° is approximately 29 nm.

### Fourier transform infrared (FTIR)

**Fig. 2:**
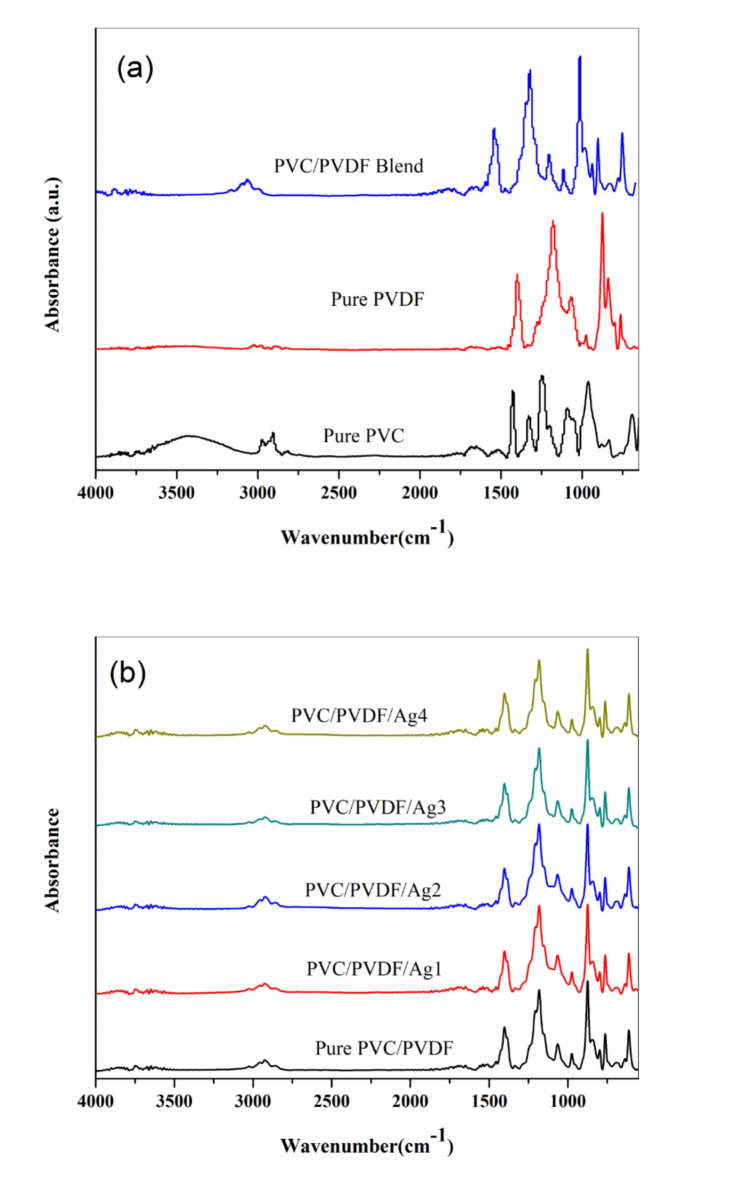
FT-IR spectra of (**a**) pure PVC, pure PVDF, and PVC/PVDF blend; (**b**) PVC/PVDF blend and PVC/PVDF with different ratios of silver nanoparticles.

IR spectra have been obtained by using FT-IR spectrophotometer. The FT-IR spectra of PVC (30)/PVDF (70) blend scattered by various concentration of Ag nanoparticles appear in the wavenumber range from 4000 nm to 400 nm. Figure 2a showed that the absorption peak of pure PVC appear at (2972-2908-1427-1249-962-834 and 609 cm^− 1^). The assignment of pure PVC obtains the bands at 2972 cm^− 1^ and 2908 cm^− 1^, which were assigned to CH_2_ asymmetric stretching vibration mode^35^. The band at 1427 cm^− 1^ was belonging to aliphatic C-H bonding. The peak at 1249 cm^− 1^ is assigned to C-H bending vibration near Cl^34^. The peak at 962 cm^− 1^ due to chain (C-C) stretching mode of PVC backbone^41^, the small band at 834 cm^− 1^ ascribed to aliphatic group C-Cl stretching mode and the sharp peak at 609 cm^− 1^ belonging to stretching vibration of the C single bond Cl^42^. The assignments of pure PVDF were obtained that the band at 1401 cm^− 1^ assigned to CH_2_ Wagging mode^43^. the peak at 1275 cm^− 1^attributed to β-phase characterization band^44^, the band at 1180 cm^− 1^ belonging to asymmetric stretching vibration of CF_2_ group^45^ the band at 1068 cm^− 1^ ascribed to CH_2_ wagging mode, β-phase band at 874 cm^− 1^^46^ due to CF_2_ symmetric stretching, α-phase properties band at 783 cm^− 1^^47^. The chemical structure of PVC is covalent bonds that cannot be broken, so no chemical reactions occur with the PVDF. FT-IR spectrum of pure PVC/PVDF blend, which is characterized by the disappearance of characteristics bands of PVC at 2972, 2908, 1427 1249 and 609 cm^− 1^ and of PDVF at 1401, 1180, and 874 cm^− 1^ and the appearance of new bands at 1540, 1318, 1202, 1115, 902, 747 cm^− 1^. This is in line with the XRD results and confirms the miscibility and robust interaction between PVDF and PVC after mixing.

Figure 2b shows FTIR absorption spectra of PVC/PVDF polymer blend doped by various contents of silver nanoparticles (AgNPs). FTIR spectra detect that characteristic bands belonging to each polymer separately shifted in some characteristic band indicated the interaction between the polymeric blend and dopant.When AgNPs have been added, a change in intensity of the fingerprint region has been obtained. A minor change in the region 200–600 cm^− 1^ was observed associated to the low content of AgNPs. The intensity of the bands decreases with increasing AgNPs concentrations. Decreasing the intensity of peaks in FTIR spectrum confirms that after increasing the concentration of AgNPs, the numbers of PVC/PVDF chains are increased in the structure of the films. The previous observations obtain the structure rearrangement between PVC/PVDF and AgNPs.

### Optical properties

**Fig. 3:**
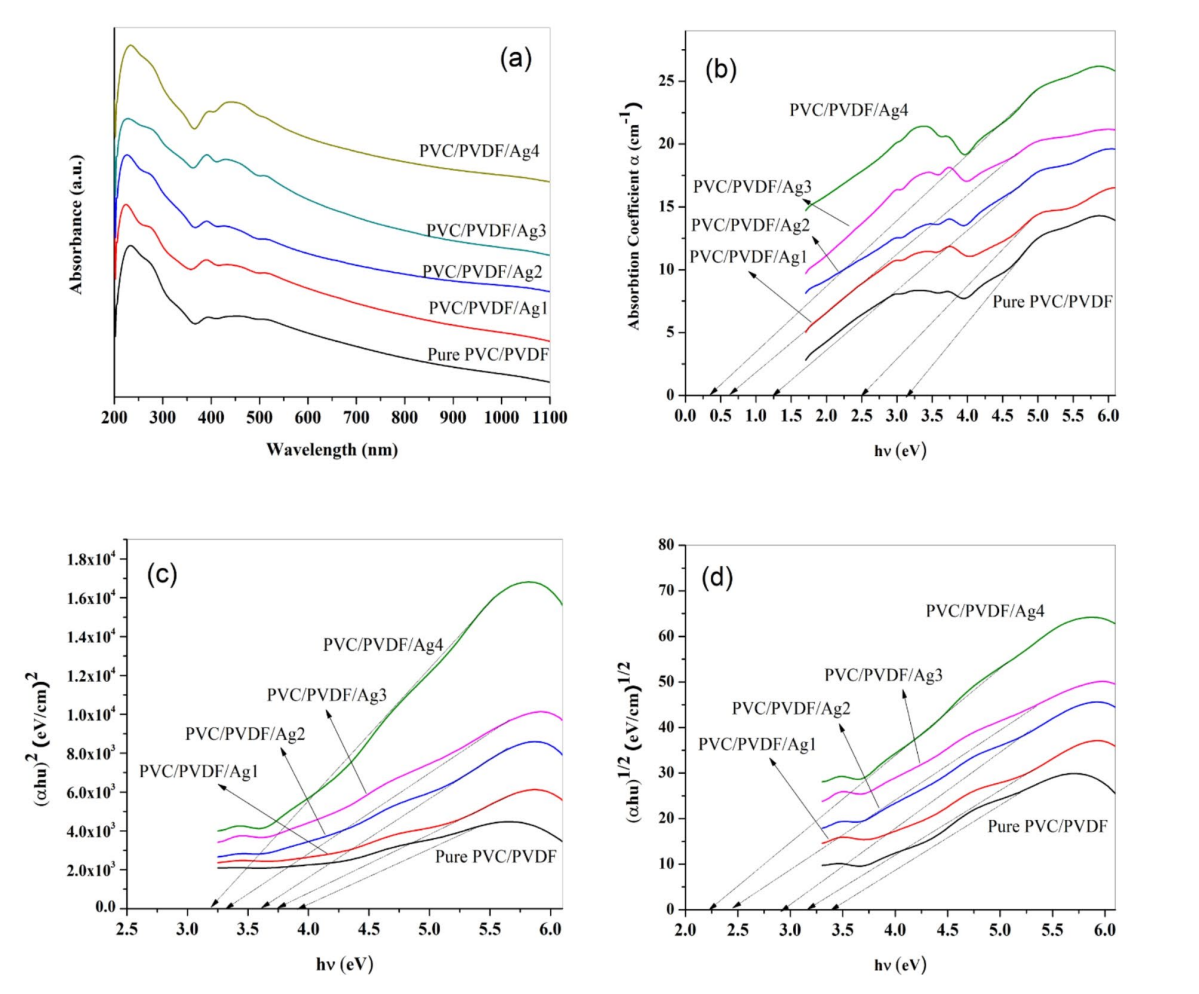
(**a**) UV-Visible absorption spectra, (**b**) plots of absorption coefficient (α) versus (hʋ); (**c**) relation between (αhʋ)^2^ versus hʋ; (**d**) relation between (αhʋ)^1/2^ versus hʋ of pure PVC/PVDF blend and PVC/PVDF with different ratios of silver nanoparticles.

UV-Visible absorption spectra of the synthesized films are collected in the range 200–1100 nm using spectrophotometer. The semicrystalline behavior of the composites showed using the absorption edges which observed at 234 nm for all of the samples. The absorption band observed at approximately 340 nm was assigned to π−π* bonding. The band appears in the range 426–454 nm belonging to silver NPs Fig. 3a. It was observed that increasing the content of AgNPs to the blend lead to small shift of the fundamental absorption edge followed by a change in the optical energy gap.

The optical energy band gap obtained by plot the relations between (αhʋ)^1/2^, (αhʋ)^2^ and energy^21,48^.

2 $$({\text{E = h}}\upsilon).$$

Calculation the absorption coefficient (α) using Beer Lambert’s Equations^48,49^ and obtained in Fig. 3b.

3 $$\alpha {\text{ }} = {\text{2}}.{\text{3}}0{\text{3 }} \times \,\frac{A}{d},$$

where A is the absorbance and d is the thickness of the film.

The values of the optical energy gap were plotted as photon energy (hʋ) verus (αhʋ)^1/2^ and (αhʋ)^2^ for both direct and indirect transition as shown in Fig. 3c,d.

The energy gap (E_g)_ obtained using Thutpalli and Tomlin method^21,48^:

4 $${\text{h}}\upsilon {\text{ }} = {\text{ }}({\text{ah}}\upsilon )^{{\text{s}}} + {\text{E}}_{{\text{g}}} .$$

The s constant has different values for different types of transition: s = 2 for direct transition, s = 1/2 for indirect transition.

**Table 2:** Values of optical parameters of PVC/PVDF matrix and PVC/PVDF with different ratios of silver nanoparticles.

Samples	Absorption edge (eV)	Direct energy bandgap (E_gd_) (eV)	Indirect energy bandgap (E_gi_) (eV)	*n*
Pure PVC/PVDF	3.15	3.95	3.41	2.24
PVC/PVDF/Ag1	2.53	3.76	3.18	2.27
PVC/PVDF/Ag2	1.26	3.63	2.94	2.37
PVC/PVDF/Ag3	0.63	3.33	2.48	2.41
PVC/PVDF/Ag4	0.33	3.21	2.21	2.46

It was obtained that absorption edge has been decreased with increasing the content of silver nanoparticles in PVC/PVDF matrix, the values of absorption edge are recorded in Table 2; it can see that the absorption edge is 3.15 eV for pure blend (PVC/PVDF) and 2.53 eV for PVC/PVDF/Ag1. The absorption edge has substantially dropped gradually by lowering the quantity of AgNPs till it reached 0.33 eV for PVC/PVDF/Ag4. The direct and indirect energy band gap values for PVC/PVDF were 3.95 eV and 3.41 eV, respectively, whereas the values for PVC/PVDF/Ag1 were 3.76 eV and 3.18 eV. Finally, it was discovered that the direct and indirect energy gap values reduce as the amount of AgNPs increases, as does the laser ablation duration, which is related to the chemical interaction between the blend and AgNPs.

The (n) refractive index used to indicate the effect of increasing the laser ablation time, determined using Dimitrov and Sakka equation in the terms of indirect energy band gap^30,50^:

5 $$\:\frac{{\text{n}}^{2\:}-1}{{\text{n}}^{2}+2}=1-\surd\:\frac{{\text{E}}_{\text{g}\text{i}}}{20}.$$

The values of *n* that are recorded in Table 2 show raising with raising in the concentration of silver nanoparticles. The observed increase in refractive index (*n*) accomplished with the decreasing in the optical energy band gap for PVC/PVDF/Ag4 in comparison with pure blend.

### Morphology and topography

**Fig. 4:**
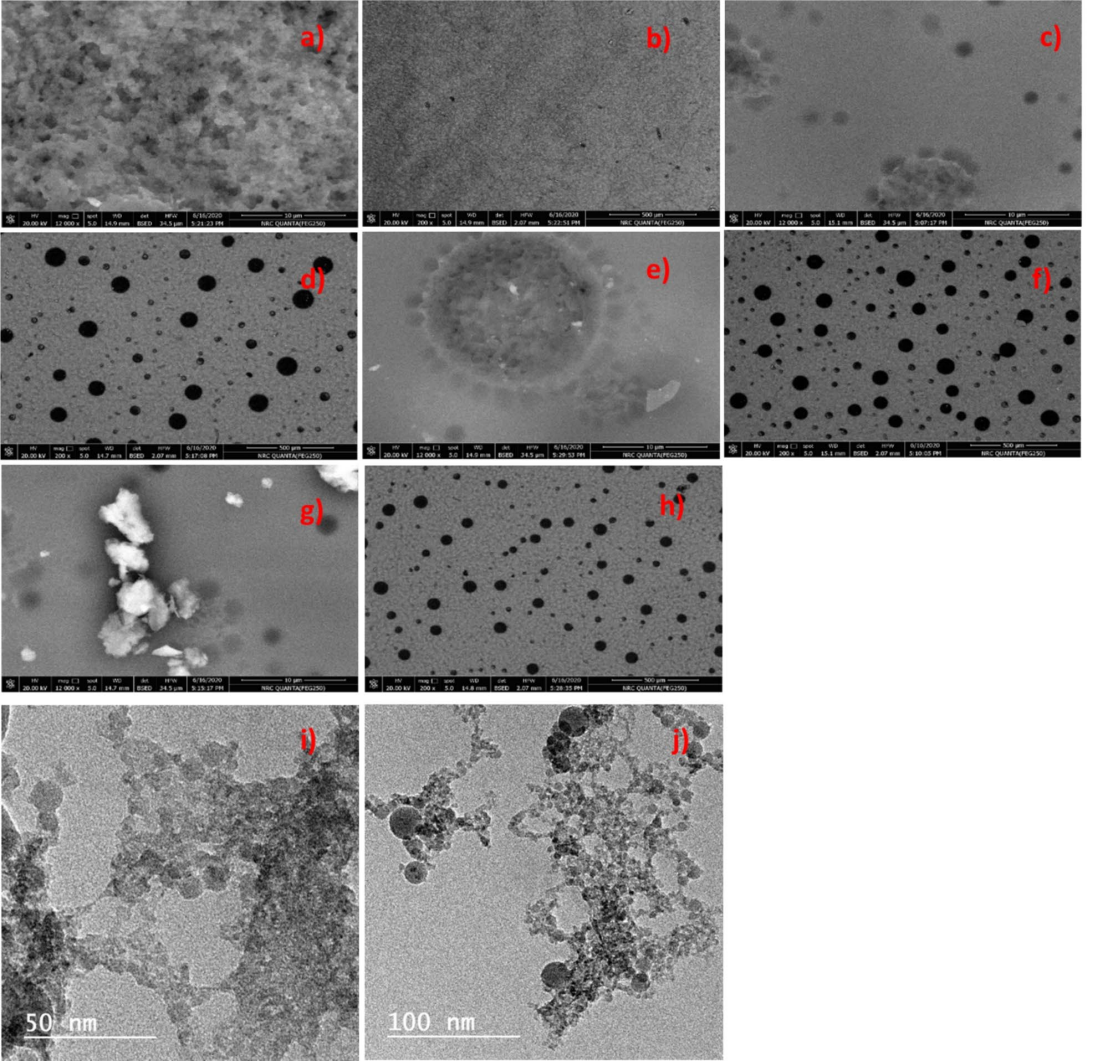
FE-SEM photos of (**a**,**b**) pure PVC/PVDF, PVC/PVDF with different ratios of silver nanoparticles; (**c**,**d**) AgNPs 1, (**e**,**f**) AgNPs 2, and (**g**,**h**) AgNPs 4, and (**i**,**j**) TEM images of AgNPs.

The morphology of the surface of and the distribution of silver nanoparticles in PVC/PVDF blend have been investigated via FE-SEM. Figure 4 shows the SEM micrograph of the surface morphology of pure PVC/PVDF blend and PVC/PVDF doped by silver nanoparticles with different ratios. Figure 4 (a,b) FE-SEM photos of the surface of pure PVDF/PVC blend illustrate a smooth homogeneous structure of the synthesized blend and that confirm the compatible between the two polymers. Figure 4c,d illustrate the low doping of silver nanoparticles (PVC/PVDF/Ag1) with PVC/PVDF polymer blend. Figure 4c showed spherical shaped of silver nanoparticles in random distribution, Fig. 4d indicate that a few nanoparticles distributed on the surface of the blend. Figure 4e,f shows SEM photos of PVC/PVDF/Ag2. Figure 4e showed spherical shape of AgNPs aggregates in PVC/PVDF blend with one large circular shape with a bright spot, Fig. 4f obtained the spherical shape of AgNPs reduced in size accompanied by an increase in number. Figure 4 (g,h) illustrates the doping of PVC/PVDF/Ag4 with PVC/PVDF blend. Figure 4e showed AgNPs aggregates on the surface and become an overlapping network, Fig. 4h indicated the number of spherical shapes belonging to silver nanoparticles increased in number with variation in size. Figure 4 from (c–h) AgNPs were aggregates in the surface and appeared as granule with different sizes. By raising the contents of AgNPs the size of aggregation decreases and become denser. These observations illustrate the complexation process between AgNPs and PVC/PVDF blend.

**Fig. 5:**
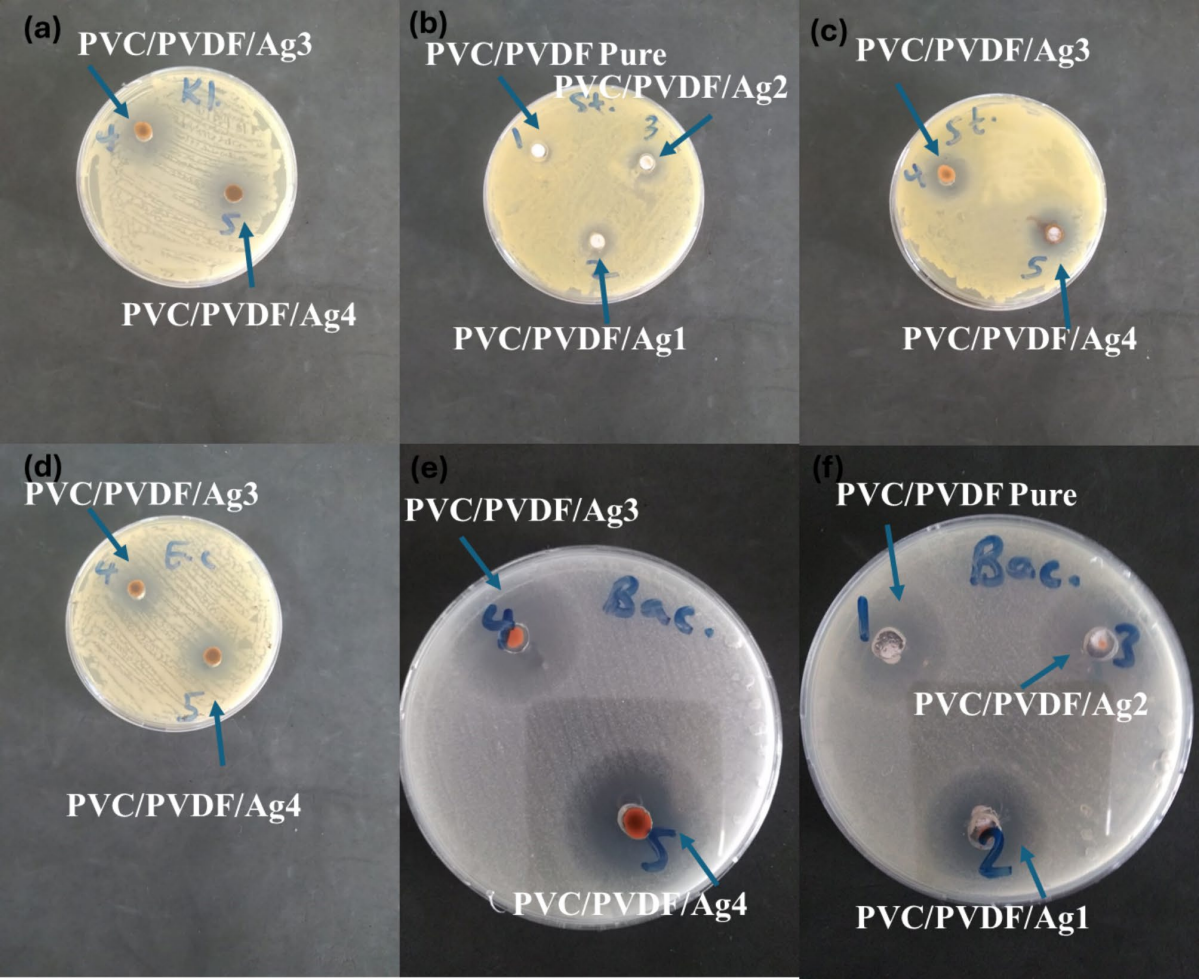
Antibacterial activity of pure PVC/PVDF blend and PVC/PVDF with different ratios of silver nanoparticles (**a**) against *Klebsiella pneumonia*, (**b**,**c**) against *Staphylococcus aureus*, (**d**) against *Escherichia coli*, and (**e**,**f**) against *Bacilus Subtits*.

Transmission electron microscopy (TEM) facilitates the imaging of AgNPs at the nanoscale, elucidating their morphology and size distribution. Figure 4 (i,j) showed that the AgNPs have spherical shape with various size, which agree with the literature^51^; in addition, it showed that the NPs are agglomerated. The particle size varies from 2.6 nm to 26.17 nm with an average particle size of 9.5 ± 5.6 nm. The diminutive particle size of AgNPs markedly improves the antibacterial and wound healing efficacy. Their distinctive attributes provide enhanced surface area and contact with biological elements, rendering them efficient in infection control and tissue regeneration promotion^52,53^.

### Antibacterial studies

The bacterial activity of PVC/PVDF doped by silver nanoparticles shows high activity index values for both gram positive and gram-negative bacteria. Increasing nanoparticles content was combined with increasing the activity index of the corresponding samples. The results in Table 3 and Fig. 5 show the variation of inhibition zone of the blend without and with adding the different concentration of AgNPs. The bactericidal activities at PVC/PVDF/Ag4 show the highest values of inhibition zone of inhibition for all the two-gram negative and for the two-gram positive. The inhibition zone values for PVC/PVDF/Ag4 are 11 ± 1, 10 ± 1, 15.7 ± 0.6, and 17.7 ± 0.6 mm for *Escherichia coli*,* Klebsiella pneumonia*,* Staphylococcus aureus and Bacillus cereus*, respectively.

In the other words, we can say that PVC/PVDF/Ag4 are valuable than the other samples, as the inhibition efficiency of the bacterial growth was enhanced. AgNPs make crosslinked with the blend (PVC/PVDF) and then accumulate and attach to the surface of the bacterial cell membrane and distribute their power function for making a hole through which the bacterial cannot passage. The binding of the nanocomposite depends on the surface area available for interaction. The higher concentrations of AgNPs having the larger surface area available for interaction will give more bactericidal effect than the lower concentration. According to the findings of the current study, silver nanoparticles may have antibacterial activity against the microorganisms found in PVC/PVDF. The findings point to PVC/PVDF/AgNPs composites as potential antibacterial materials for wound healing.

**Table 3:** The antibacterial activity against two-gram negative bacteria and against two gram-positive of pure PVC/PVDF blend and PVC/PVDF with different ratios of silver nanoparticles.

Microorganism	Sample					
	PVC/PVDF pure	PVC/PVDF/Ag1	PVC/PVDF/Ag2	PVC/PVDF/Ag3	PVC/PVDF/Ag4	Standard antibiotic
Gram negative bacteria						Gentamicin
*Escherichia coli (ATCC:10536)*	NA	NA	NA	11.0 ± 1.0	11.0 ± 1.0	27.3 ± 0.6
*Klebsiella pneumonia (ATCC:10031)*	NA	NA	NA	9.3 ± 0.6	10.0 ± 1.0	28.0 ± 1.0
Gram positive bacteria						Ampicillin
*Staphylococcus aureus (ATCC:13565)*	9.7 ± 0.6	9.7 ± 0.6	12.0 ± 1.0	12.7 ± 0.6	15.7 ± 0.6	21.3 ± 0.6
*Bacilus Subtits (DSM:1088)*	13.7 ± 0.6	14.7 ± 0.6	15.7 ± 0.6	16.3 ± 0.6	17.7 ± 0.6	21.7 ± 0.6

The results of the One-Way ANOVA presented in Table 4 reveal statistically significant differences in antimicrobial efficacy between the different PVC/PVDF/Ag composites (Ag3, Ag4) and the standard antibiotic for all tested microorganisms. For *Escherichia coli*, the F-value of 265.69 (*p* = 0.011) suggests a highly significant variation among the groups, indicating that the addition of silver (Ag) to the PVC/PVDF matrix significantly enhances antimicrobial activity compared to the control. Similarly, *Klebsiella pneumonia* shows a substantial F-value of 337.09 (*p* = 0.016), further supporting the superior efficacy of the Ag-modified materials over the standard treatment. For the Gram-positive bacteria, *Staphylococcus aureus* demonstrated significant differences with an F-value of 57.16 (*p* = 0.025), implying that the Ag4 composite is markedly more effective than both Ag3 and the control. The lowest but still highly significant F-value of 23.56 (*p* = 0.033) for *Bacillus subtilis* also confirms that silver-enhanced composites exhibit superior antimicrobial performance. Overall, these results suggest that the incorporation of silver into the PVC/PVDF matrix substantially improves antimicrobial properties, making Ag3 and Ag4 promising materials for antibacterial applications, particularly against Gram-negative bacteria.

**Table 4:** Results of one-way ANOVA comparing the antimicrobial efficacy of PVC/PVDF/Ag3, PVC/PVDF/Ag4, and the standard antibiotic against four different microorganisms. Statistically significant differences were found for all microorganisms (*p* < 0.05).

Microorganism	F-value	*p*-value
*Escherichia coli*	265.69	0.016
*Klebsiella pneumonia*	337.09	0.011
*Staphylococcus aureus*	57.16	0.025
*Bacillus Subtilis*	23.56	0.033

The diminutive particle size of AgNPs markedly improves their antibacterial and wound healing efficacy. Their distinctive attributes enhance surface area and interaction with biological elements, rendering them helpful in infection control and tissue regeneration promotion. Smaller AgNPs enhance tissue penetration, hence improving their efficacy in promoting wound healing^54^. Research indicates that AgNPs can be integrated into wound dressings, offering a protective barrier and facilitating healing processes^52,55^.

### Cell viability

**Fig. 6:**
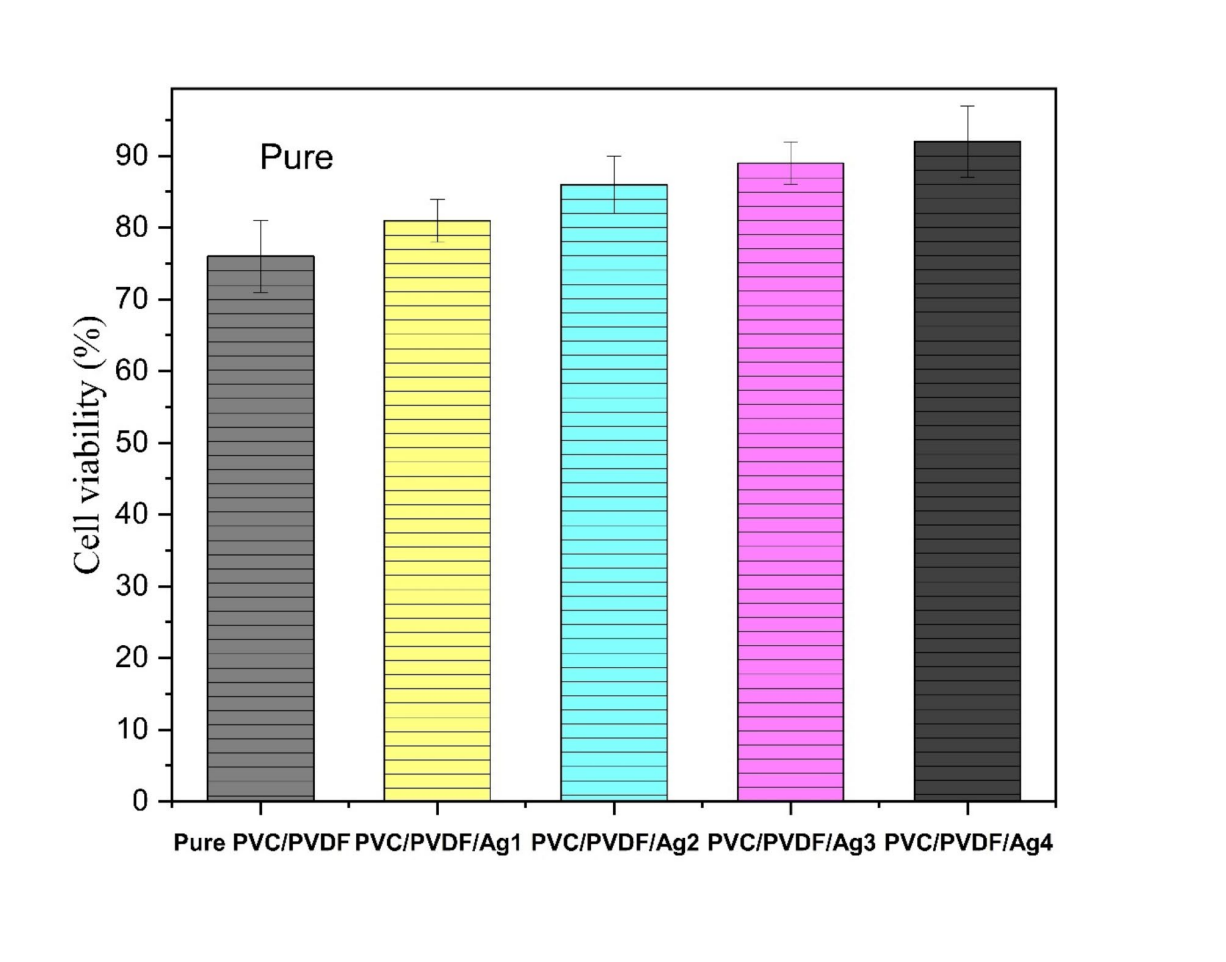
The cell viability test for pure PVC/PVDF membrane and PVC/PVDF with different concentrations of silver nanoparticles.

The cell viability test is a crucial test to illustrate whether the material is toxic against human cells or not. The prepared nanocomposites showed a gradual increase in cell viability percentage by increasing the concentration of Ag NPs in the polymeric matrix as shown in Fig. 6. All membranes are tested at the same concentration, which is a high concentration, against human fibroblast cell line to study its viability in wound healing applications. The results showed that the pure PVC/PVDF has cell viability of 76 ± 5%; the addition of Ag NPs showed a significant increase in this percentage for example, the PVC/PVDF/Ag1 membrane showed viability of 81 ± 3%, the PVC/PVDF/Ag2 membrane exhibited a viability of 86 ± 4%, the PVC/PVDF/Ag3 membrane showed a viability of 89 ± 3%, and the highest cell viability obtained in the membrane of PVC/PVDF/Ag4 which has the highest content of Ag NPs with a viability of 92 ± 5%. It could be noticed that all membranes have a cell viability higher than the IC_50_ limit at high concentrations which means that the membranes are not toxic and safe to be used in wound healing applications. Studies indicate that Ag NPs can be biocompatible at certain concentrations, promoting cell proliferation without significant cytotoxic effects^56^. Ag NPs exhibit strong antibacterial activity, which reduces microbial contamination and promotes a favorable environment for cell growth^57,58^. The antibacterial results agree with this claim which means that the higher the concentration of the AgNPs the higher the antibacterial activity and better cell proliferation.

## Conclusion

Nanocomposite films consist of Polyvinylidene fluoride (PVDF)/polyvinyl chloride (PVC) blend doped with different concentrations of silver nanoparticles (Ag) were prepared and the films were studied using different techniques. The polymer blend prepared by the traditional solution casting technique. nanoparticles obtained using laser ablation technique in liquid. IR analysis reveals that no new peaks appear after adding silver nanoparticles, the characteristic band of single polymer shifted in some characteristic bands these bands intensity was observed in the range 200–600 cm^− 1^, the intensity of the bands decrease with increasing the concentration of silver nanoparticles. X-ray diffraction indicating that the peak at 2θ = 38.42̊ belonging to the high concentration of Ag nanoparticles thus the amorphous nature of the blend increased. From UV-Visible analysis, the absorption edges observed at 234 nm for all of the samples, the band appears in the range 426–454 nm belonging to AgNPs. The band-gap energy obtained from UV/Vis measurements decreased with increasing Ag content due to the chemical bonding existing between the blend and silver nanoparticles. SEM illustrated increasing the concentration of silver nanoparticles (AgNPs) the size of spot decrease and the aggregation become denser. All measurements confirmed the interactions that occurred between PVC/PVDF and AgNPs. The inhibition efficiency of the bacterial growth was enhanced after adding silver nanoparticles. It was observed that gram-positive bacteria more affected by AgNPs than gram-negative bacteria. The main idea of the research is improvement antibacterial activity of the blend by using silver nanoparticles prepared by laser ablation. Antibacterial activity was inhibited at pure blend while becoming more active at the higher concentration of silver nanoparticles. The research evaluated the biocompatibility of nanocomposite membranes containing silver nanoparticles (Ag NPs) in a polymeric matrix. Increasing Ag NP concentrations led to higher cell viability percentages, particularly beneficial for wound healing applications on human fibroblast cell lines. Membranes with higher Ag NP content demonstrated elevated cell viability compared to pure PVC/PVDF, with viability percentages ranging from 81 to 92%. All membranes surpassed the IC50 toxicity limit at high concentrations, indicating their non-toxicity and suitability for wound healing applications.

## Data Availability

The datasets generated during and/or analysed during the current study are available from the corresponding author on reasonable request.
